# Evaluation of the Bioavailability of Iodine and Arsenic in Raw and Cooked *Saccharina japonica* Based on Simulated Digestion/Caco-2 Cell Model

**DOI:** 10.3390/foods13182864

**Published:** 2024-09-10

**Authors:** Na Li, Zhaomeng Geng, Yingying Guo, Xinyue Dai, Wenjia Zhu, Lin Yao, Yanhua Jiang, Xiaojuan Wang, Hao Dong, Huijie Wang, Lianzhu Wang

**Affiliations:** 1 Yellow Sea Fisheries Research Institute, Chinese Academy of Fishery Sciences, Qingdao 266071, China; lina251821@163.com (N.L.); wanglz@ysfri.ac.cn (L.W.); 2 College of Food Science and Engineering, Ocean University of China, Qingdao 266003, China; 3 College of Food Science, Dalian Polytechnic University, Dalian 116034, China; 4 Shandong Meijia Group Co., Ltd., Rizhao 276800, China

**Keywords:** *Saccharina japonica*, bioaccessibility, bioavailability, iodine, arsenic

## Abstract

Kelp is a traditional healthy food due to its high nutritional content; however, its relatively high contents of iodine and arsenic have raised concerns about its edible safety. This study explored the effects of different cooking treatments on the contents of iodine and arsenic in kelp, evaluated the bioaccessibility and bioavailability of iodine and arsenic in kelp using in vitro digestion, and compared the differences in the transport characteristics of iodine in kelp and KIO_3_ using a Caco-2 monolayer cell transport model. The results show that the content of target elements that reached systemic circulation could be reduced by cooking and gastrointestinal digestion. The highest reductions in iodine and arsenic were 94.4% and 74.7%, respectively, which were achieved by boiling for 10 min. The bioaccessibility and bioavailability of iodine and arsenic were significantly improved by a cooking treatment. However, the contents of iodine and arsenic decreased significantly, with the bioaccessibility of iodine reducing from 3188.2 μg/L to 317.0 μg/L and that of arsenic reducing from 32.5 μg/L to 18.1 μg/L in the gastric phase after boiling. The findings also show that the efficiency of iodine transport in kelp and KIO_3_ was positively correlated with the transport time and negatively correlated with the concentration of iodine. With the increase in the iodine concentration, the rate of iodine transport in kelp decreased from 63.93% to 3.14%, but that of KIO_3_ was stable at around 35%, which indicates that the absorption efficiency of iodine from kelp was limited, even when too much kelp was ingested. In conclusion, the edible safety of kelp is significantly improved after cooking. The risk of excessive iodine and arsenic intake caused by consuming kelp is extremely low, and as an effective iodine supplement source, kelp has higher edible safety compared with KIO_3_. This study clarifies the safety of algae based on iodine and arsenic contents and also provides a basis for the formulation of food safety standards.

## 1. Introduction 

Kelp (*Saccharina japonica*), a brown seaweed, contains multiple nutritionally beneficial compounds and is widely used not only for human consumption but also in feed, fertilizer, and brown alginate industries. It forms a complete chain from breeding and processing to application [1]. Kelp is rich in minerals, and its consumption promotes the intake of beneficial essential elements such as iodine, selenium, and zinc [2]. Therefore, kelp is an ideal source of marine plant-based nutrients [3,4]. However, it is important to note that kelp may also contain relatively high concentrations of potentially toxic elements such as iodine and arsenic, which may cause food safety risks and health concerns resulting from its consumption [5,6]. 

Iodine (I) is an essential element in humans that is necessary for the synthesis of thyroid hormones and the regulation of human growth and development, especially in the early stages of life [7]. However, excessive intake of I can adversely affect human health by causing thyroid dysfunction and increasing the risk of hypo- and hyperthyroidism [8,9,10]. The tolerable upper intake levels (ULs) of iodine have been established by different countries depending on the basal status of iodine intake and age. For instance, the ULs for iodine set by the National Health and Family Planning Commission of PRC (NHFPCPRC) are 200 μg of iodine/day for children 4–6 years of age, 300 μg of iodine/day for boys and girls 7 to 10 years of age, 400 μg of iodine/day for boys and girls 11 to 13 years old, 500 μg of iodine/day for teenagers 14 to 17 years old, and 600 μg of iodine/day for adults [11]. Arsenic (As) is a toxic metalloid due to its detrimental effects on human health, with the toxicity of As depending on its chemical forms. As exists in inorganic and organic forms, with inorganic arsenic being more toxic [12,13]. The long-term intake of inorganic As can increase the risk of cancer of the liver, bladder, lungs, and skin [14]. Relevant research has shown that the major chemical form of As in kelp is the organic form, which is considered non-toxic to humans [15,16].

Some brown algae, especially Saccharina spp., have extremely high levels of iodine, in some cases exceeding 10,000 mg/kg dw [17]. Kelp is the species with the highest reported mean iodine concentration of 3529 mg/kg [18]. In particular, in Asian countries, the ingestion of seaweed soup as a part of the daily diet is common. The average consumption of seaweed has been estimated to be 5.2 g/adult per day among Chinese individuals, 10.4 g per day among Japanese individuals, and 8.5 g per day among South Korean individuals [19,20]; however, even with this high iodine intake from kelp, the incidence of iodine-induced health problems does not appear to be severely elevated in Asian countries. 

Raw kelp is not suitable for eating and needs to undergo a cooking treatment such as blanching, boiling, steaming, etc., before being eaten. During the process of cooking, the levels of I and As decrease to a certain extent [21]. After kelp is ingested, it undergoes digestion in the stomach and intestines. The digested kelp is then absorbed and accumulates in the tissues and organs along with the blood, which can affect the quantity of elements that ultimately reach systemic circulation [22]. Therefore, it is important to understand how much of the iodine and arsenic in kelp can be released into the gastrointestinal environment and how much of the released iodine and arsenic is absorbed by the body. A simulated in vitro digestion model could provide an effective approximation of in vivo situations and offer the advantages of reproducibility [23]. The Caco-2 monolayer cell transport model facilitates the evaluation of intestinal cell retention and transport processes and offers a more reliable approximation for in vivo situations in estimating the bioavailability of elements at the intestinal level [24]. While some work has been carried out in this field, there is limited information available on the difference in transport characteristics between iodine in kelp and inorganic iodine, which plays an important role in clarifying the dietary safety of iodine from kelp sources [25]. Therefore, the aim of the present study was to clarify the bioavailability of iodine and arsenic in raw kelp and kelp samples subjected to different cooking methods using a combination of an in vitro digestion model and the human epithelial colorectal adenocarcinoma cell line (Caco-2) cell model. Additionally, the transport and absorption characteristics of the two elements in the intestinal tract were investigated to objectively evaluate the edible safety of iodine and arsenic in seaweed, provide a scientific basis for the appropriate dietary intake of seaweed, and promote the formulation of food safety standards in terms of the maximum levels (MLs) of iodine and arsenic recommended in seaweed and seaweed-based products.

## 2. Materials and Methods

### 2.1. Reagents and Materials

Fresh kelp was collected from Ailian Bay in Rongcheng city, Shandong Province, and the variety is Xunshan No.1. Standard solutions for gastric and intestinal digestion were purchased from Shanghai Yuanye Bio-Technology Co., Ltd. (Shanghai, China). Bromine water 30% (m/m), potassium iodide (KI), potassium iodate (KIO_3_), sodium carbonate (Na_2_CO_3_) concentrated sulfuric acid, and sodium thiosulfate were from Sinopharm Group Chemical Reagent Co., Ltd. (Shanghai, China). Caoco-2 cells were acquired from Procell Life Science & Technology Co., Ltd. (Wuhan, China). Fetal bovine serum (FBS), Phosphate-Buffered Saline (PBS (1×)) and Dulbecco’s Minimum Essential Medium (DMEM) were acquired from Thermo Fisher Scientific Co. (Waltham, MA, USA). The alkaline phosphatase (AKP) kit was purchased from Beyotime Biotechnology Co. (Shanghai, China). Other chemicals and reagents used were of analytical grade.

### 2.2. Instrumentation

Ultra-pure water was obtained from the Milli-Q water purification system. Total iodine contents were determined by using a microwave digestion system (GEM, MASS 6 CLASSIC, Matthews, NC, USA) and an inductively coupled plasma mass spectroscopy (ICP-MS) system (Perkin Elmer, ELAN DRC II, Waltham, MA, USA). Transepithelial electrical resistance (TEER) was tested with a transmembrane resistance meter (World Precision Instruments Inc. EVOM2, Sarasota, FL, USA).

### 2.3. Bioaccessibility Assay

#### 2.3.1. Pretreatment of Kelp

The materials of in vitro simulation were divided into three groups: raw materials, boiled processing materials and steamed processing materials. The different processing methods were as follows. The kelp in the raw materials group was cut into 0.5 cm × 0.5 cm chips after being washed without any heating treatment. The kelp in the boiled processing materials group was cut into 0.5 cm × 0.5 cm chips after being washed and treated in boiling water for 10 min. The kelp in the steamed processing materials group was cut into 0.5 cm × 0.5 cm chips after being washed and treated with steaming for 10 min. All of the samples were maintained at 4 °C before digestion and the in vitro digestion should be completed in 3 days [23].

#### 2.3.2. In Vitro Digestion Procedure

Raw and cooked samples were digested using two in vitro methods, including a physiologically based extraction test (PBET) and a unified barge method (UBM). Samples were divided into 3 groups for the PBET model and 4 groups for the UBM model; each group had 2 g samples and 3 parallel pairs. Specific operations of different methods were performed according to Table 1. The contents of I and As of raw and cooked materials were detected, as well as the digestive juices in the gastric and intestinal stages in two models.

#### 2.3.3. Determination of Total Arsenic

The content of total arsenic in digestive juice was determined using an ICP-MS system. Briefly, about 1.0 mL of sample was completely decomposed with 5 mL of 7 M HNO_3_ for 1 h using microwave-assisted digestion. Subsequently, the digested solution was diluted to the appropriate volume with pure water (according to the total arsenic concentration in the sample), and the concentration of total arsenic in the final solution was detected [29,30].

#### 2.3.4. Determination of Total Iodine

The content of total iodine in digestive juice was determined by the ICP-MS system. In total, 1.0 mL of sample was added to 5.0 mL of 4% (CH_3_)_4_NOH in a centrifuge tube of 50 mL. After vortex blending for 1 h and incubating in a water bath shaker at 85 ± 5 °C for 3 h, the mixture was cooled and filled to 50 mL with water. The samples were centrifuged at 4000 rpm for 10 min and filtered with 0.45 μm membrane. The concentration of total iodine in filtrate was detected [31]. Linearity was verified by obtaining ICP-MS measurements of iodine standards (0, 1, 5, 10, 15, 20, 50 and 100 µg/L). The limits of detection and quantification (LOD and LOQ, respectively) were determined according to the results obtained from the method blanks. 

The content of total iodine in kelp was determined by oxidation–reduction titration. In total, 2 g of kelp was added to 5 mL NaCO_3_ solution and dried. And then the samples were carbonized and burned in a muffle furnace at 550 °C ± 25 °C for 40 min. After cooling, the samples were ground with water and transferred into a beaker of 250 mL, boiling for 5 min and filtered to an iodine flask of 250 mL. In total, 3 drops of methyl orange solution were added and the system was changed into red with 1M sulfuric acid solution, supplemented with 5 mL of saturated bromine water and the solution was boiled until the yellow disappeared. Subsequently, 5 mL of sodium formate solution was added, boiling for 2 min. The solution was cooled in water bath to below 30 °C and then supplemented with 5 mL of 3 M sulfuric acid solution and 5 mL of potassium iodide solution. The mixture above was titrated with sodium thiosulfate standard solution to yellow and then added to 1 mL of starch solution. We continued to titrate until the blue color disappeared.

#### 2.3.5. Calculation of Bioaccessibility

The bioaccessibility of raw and cooked samples was defined as the proportion of total content of I and As in materials for absorption, and it was calculated by the formula below:

$$Bioaccessibility(\%)=\frac{\left(I\;or\;As\;in\;bioaccessible\;fraction\right)}{\left(I\;or\;As\;in\;materials\right)}\times 100$$

### 2.4. Bioavailability and Transport Assay

#### 2.4.1. Establishment of Caco-2 Monolayer Cell Transport Model

Caco-2 cells were cultured in a humidified atmosphere with 5% CO_2_ at 37 °C in DMEM medium supplemented with 10% FBS and 1% P/S. The passage numbers of experimental cells were between ten and twenty. Cells exhibiting healthy morphology and high growth potential, reaching approximately 70–80% confluence, were selected for the experiments. Logarithmic phase cells with three different concentrations of 7 × 10^4^/mL, 12 × 10^4^/mL and 20 × 10^4^/mL were inoculated into transwell plates: the apical side (AP) of the membrane was added to 1000 µL of cell suspension and the basolateral side (BL) of the membrane was added to 1500 µL of free medium. The cells were cultured continuously for 21 days and the supernatant should be replaced every two days. The Caco-2 monolayer cell transport model was established by monitoring alkaline phosphatase (AKP) activity, transmembrane resistance value (TEER) and surface microvilli structure as indicators [32,33].

#### 2.4.2. Detection of Activity of Alkaline Phosphatase (AKP)

The biochemical characteristics and cell polarity of Caco-2 cells were determined by measuring the activity of AKP in each stage of the Caco-2 cell layer. Samples were collected from both sides of the transwell membrane on the 3rd, 6th, 9th, 12th, 15th and 21st days for AKP activity measurement [34].

#### 2.4.3. Detection of Transepithelial Electrical Resistance (TEER)

The resistance values of cells in the inoculated plate were detected on the 3rd, 6th, 9th, 12th, 15th and 21st days. The culture solution was discarded, and the cells were cleaned with preheated PBS solution three times. For the last time, the cells were incubated for 30 min before measuring TEER [35]. 

#### 2.4.4. Morphological Observation

Scanning electron microscope (SEM): The Caco-2 cells that met the requirements of AKP and TEER were fixed with 5% glutaraldehyde for 24 h. Subsequently, the cells along with the polycarbonate membrane were removed and cut into rectangles measuring 0.5 cm × 0.8 cm. After dehydration and gold spraying, the cell morphology and microvilli were observed using scanning electron microscopy [36].

Transmission electron microscope (TEM): The Caco-2 cells that met the requirements of AKP and TEER were fixed with 5% glutaraldehyde for 24 h, and the cells along with the polycarbonate membrane were removed and cut into small squares, and made into ultra-thin sections after dehydration and embedding. The differentiation of cells was observed via transmission electron microscopy. 

#### 2.4.5. Calculation of Bioavailability

The intestinal digestive solution was filtered through a 0.22 μm membrane for reserve. After the cell model was completed, the medium in the transwell chamber was removed and washed with PBS; then, 2000 µL of intestinal digestive solution in the upper chamber and 2000 µL of buffer in the lower chamber, the same molar concentration of KIO_3_, were used as the control group. The contents of iodine and arsenic in the upper chamber and lower chamber were detected after the samples were incubated at 37 °C and 5% CO_2_ for 2 h. The bioavailability was calculated according to the following formula:

$$Bioavailability(\%)=\frac{\left(I\;or\;As\;in\;bioavailable\;fraction\right)}{\left(I\;or\;As\;in\;materials\right)}\times 100$$

### 2.5. Statistical Analysis

All of the data are given as mean ± standard deviation (SD). Statistical significance was tested by employing analysis of variance (ANOVA). Before one-way ANOVA, the normality and homoscedasticity were checked by a Kolmogorov–Smirnov test and F test, respectively. 

## 3. Results

### 3.1. Total Content of I and As in Raw and Cooked Samples

**Figure 1:**
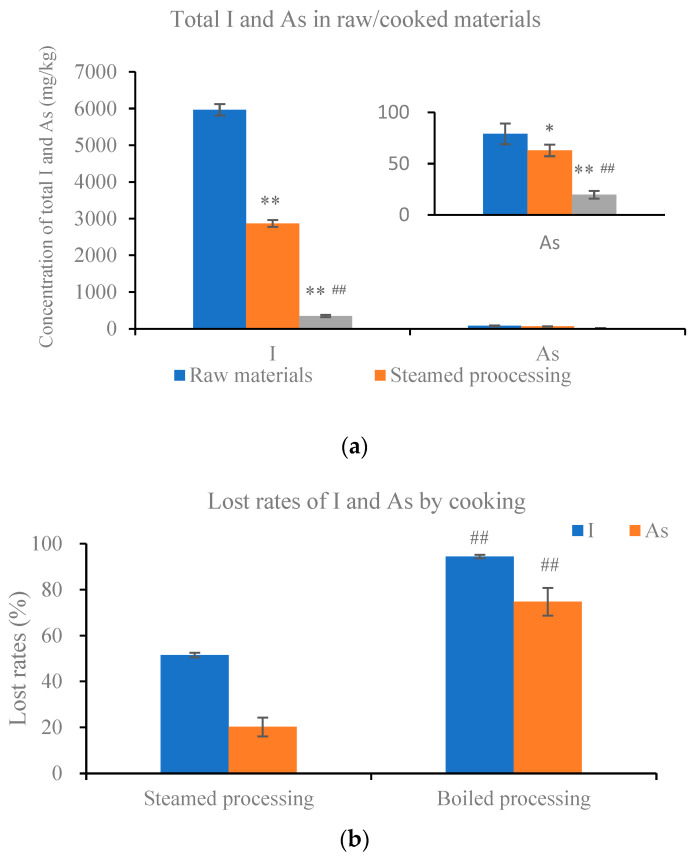
The change in iodine and arsenic by steaming and boiling. (**a**) Content of total I and As in raw/cooked kelp. (**b**) Lost rates of I and As in kelp by cooking. Values are mean ± standard error (*n* = 3). * and ** mean *p* < 0.05 and *p* < 0.01 compared with raw materials; ## means *p* < 0.01 compared with steamed processing.

To investigate the impact of cooking on the loss of iodine and arsenic, the contents of I and As in raw and cooked kelp samples were detected and analyzed, with cooking processes including boiling and steaming. The results presented in Figure 1 are based on dry weight. The original content of I in raw fresh kelp was 5964.44 ± 155.68 mg/kg, and the content of As was 79.11 ± 10.10 mg/kg. Compared with raw materials, the content of iodine and arsenic in cooked samples decreased, particularly in the processing of boiling treatment. After 10 min of boiling treatment, 94.42 ± 0.74% of I and 74.71 ± 6.07% of As were lost, respectively. In the processing of steaming, the lost rates of I and As were 51.54 ± 1.03% and 20.24 ± 4.10%, respectively. These findings indicated that cooking can lead to significant losses of iodine and arsenic, but the extent of loss depends on the cooking method.

### 3.2. Bioaccessibility of I and As in Raw/Cooked Kelp

In this study, the bioaccessibility of iodine and arsenic in fresh kelp before and after cooking was studied using two in vitro simulated digestion techniques, PBET and UBM. The results presented in Table 2 demonstrate that both steaming and boiling could enhance the bioaccessibility of iodine and arsenic. In the two digestion models, the bioaccessibility of iodine in fresh kelp was found to be 59.86 ± 5.66% and 64.63 ± 4.60%, and for arsenic, it was 48.16 ± 3.38% and 50.73 ± 4.11%, respectively. After steaming treatment, the bioaccessibility of iodine and arsenic reached 65.49 ± 4.80%~69.53 ± 6.34% and 53.03 ± 4.68%~57.74 ± 4.74%, respectively. After boiling treatment, it reached 68.03~80.21% and 59.91~61.37%, respectively. Compared to boiling treatment, steaming treatment had less effect on the bioaccessibility of iodine and arsenic in kelp.

**Figure 2:**
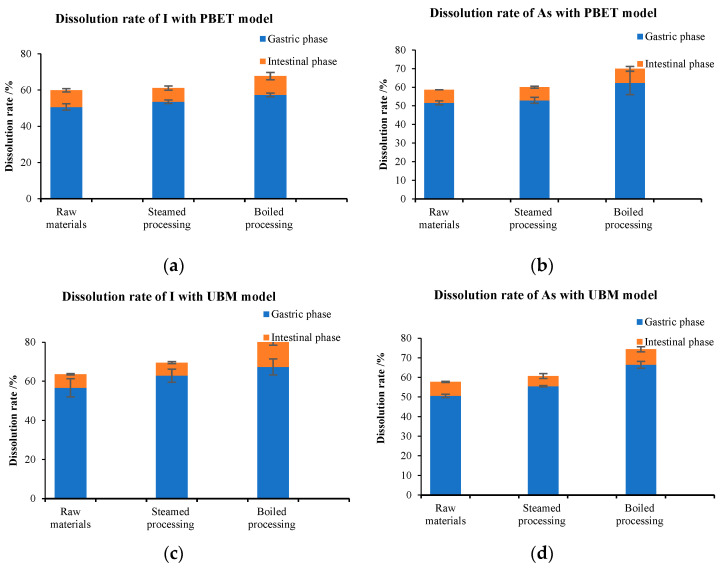
Bioaccessibility of total I and As of raw/cooked kelp. Values are mean ± SD (*n* = 3); (**a**) bioaccessibility of total I with PBET model; (**b**) bioaccessibility of total As with PBET model; (**c**) bioaccessibility of total I with UBM model; (**d**) bioaccessibility of total As with UBM model.

The dissolution rates of two elements at different stages of digestion are illustrated in Figure 2. It was observed that these elements were primarily dissolved during the gastric digestion stage and partially dissolved during the intestinal and colon stages. In the digestion model of PBET, the dissolution rates of iodine and arsenic exhibited a significant increase during the intestinal and colon phases. Furthermore, cooking treatment led to an elevation in the bioaccessibility of iodine and arsenic. In addition, disparities were observed in the bioaccessibility of these two elements in two digestion models. For instance, the dissolution rates of the two elements during the stomach digestion stage in the UBM model were generally higher than in the PBET model. This difference may be attributed to the addition of saliva components during the gastric digestion stage of the UBM method, which made it easier for the elements to dissolve compared to the PBET method.

Although cooking treatment could enhance the bioaccessibility of iodine and arsenic in kelp, it also led to a significant loss of these elements and reduced their absolute concentrations. As presented in Table 3, the absolute concentrations of iodine and arsenic in the digestive solution decrease significantly at each stage after cooking. Specifically, the total iodine content in the gastric digestive juices of boiled kelp was only 12.0% of that in fresh kelp, and the arsenic content was 12.8%. Similarly, the total concentrations of dissolved elements at each stage of the boiling and steaming treatment groups were also significantly lower than that of the raw material group. These results indicated that although cooking treatment increased the bioaccessibility of iodine and arsenic in kelp compared to uncooked samples, it decreased the bioaccessible contents of these elements significantly. Therefore, cooking treatment was conducive to improving the edible safety of kelp food.

### 3.3. The Integrity of Caco-2 Monolayer

#### 3.3.1. Alkaline Phosphatase Activity of Caco-2 Monolayer

**Figure 3:**
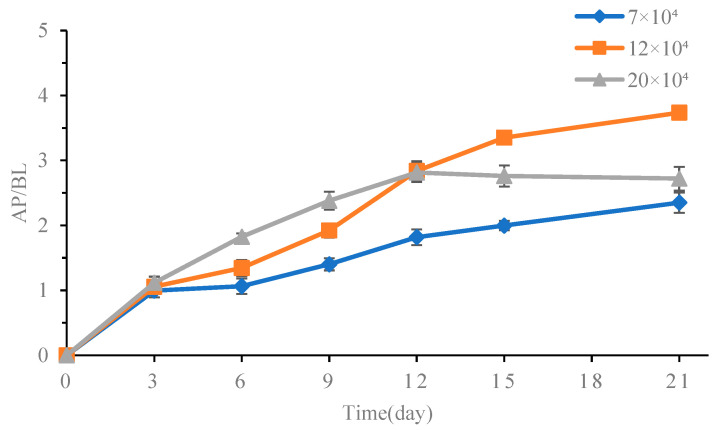
Alkaline phosphatase activity of Caco-2 monolayer. Values are mean ± standard error (*n* = 3).

Alkaline phosphatase is a prototypical enzyme of the small intestinal epithelium, which can be isolated during the formation of a single layer of Caco-2 cells [37]. The degree of cell differentiation can be determined by measuring the activity of alkaline phosphatase at both the apical side and basolateral chamber of the transwell at different time points. The results in Figure 3 show the AP/BL values for alkaline phosphatases during the modeling process using three different concentrations of cells. In the low-concentration group, cell growth was slow, with an AP/BL value still below 3.0 on day 21. The cells in high-concentration group showed rapid growth in the early stage, reaching a peak after 12 days and then declining in the later stage. This suggests that rapid proliferation in a short period is not conducive to the formation of microvillus cell structure. At the cell concentration of 12 × 10^4^ cells/mL, there was a faster increase rate in alkaline phosphatase activity was faster from the 7th to 15th day. On the 15th day, the AP/BL value of alkaline reached 3.35, indicating that the Caco-2 monolayer had gradually been differentiated and the two sides of the model had a large polarity. The AP/BL value reached 3.74 on the 21st day, indicating that both sides of the monolayer model had high polarity and could meet the transport requirements of various enzymes and vectors. The cell model was successfully established and could be used for transport experiments.

#### 3.3.2. The TEER of Caco-2 Monolayer

**Figure 4:**
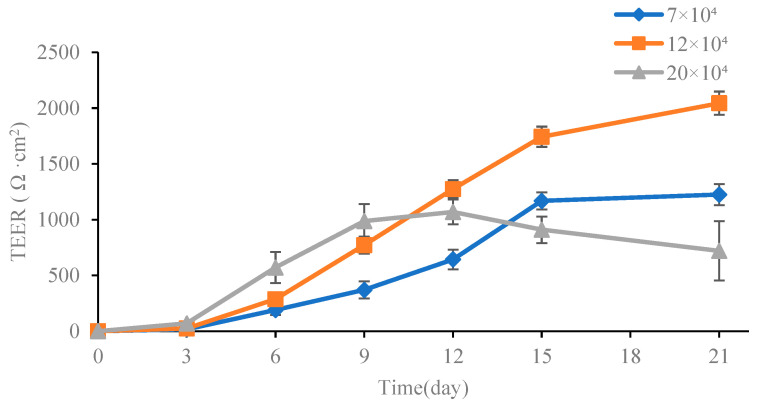
The TEER of Caco-2 monolayer. Values are mean ± standard error (*n* = 3).

The transmembrane resistance value is a typical indicator evaluating the integrity of the Caco-2 monolayer cell model [38]. A higher transmembrane resistance value indicates tighter intercellular connections. During the modeling process, the resistance values were recorded and the changes in resistance over time under three different cell concentrations are presented in Figure 4. It was observed that at the early stage, the TEER value was lower, indicating the low cell proliferation rate and large intercellular space. However, as the culture time increased, the TEER value showed an upward trend. The peak of TEER value in the high-concentration group appeared on day 12 after inoculation, while in the low-concentration group, it appeared on day 15. Nevertheless, when combined with the results of alkaline phosphatase activity, both AP/BL values were below 3.0, suggesting that the intercellular density did not meet the required standards. At the cell concentration of 12 × 10^4^ cells/mL, the TEER value increased steadily from day 7 to day 15, and reached 953 Ω·cm^2^ on day 15, indicating an increase in the degree of intercellular fusion and the formation of a relatively complete cell membrane. Subsequently, growth tended to be gradual with TEER value increasing to 1328 Ω·cm^2^ on day 20, indicating that the intercellular space was completely fused, forming a dense cell membrane. The cell model was successfully established and could be used for subsequent transport tests.

#### 3.3.3. Morphological Observation of Caco-2 Monolayer

**Figure 5:**
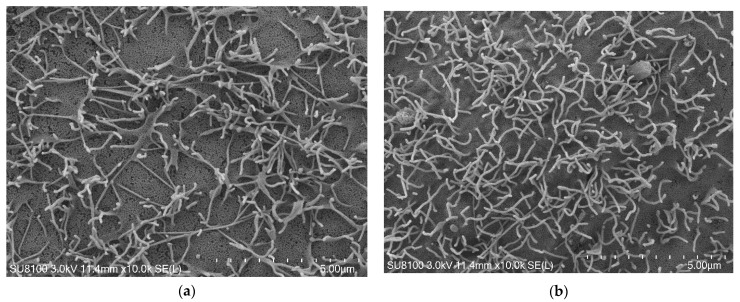

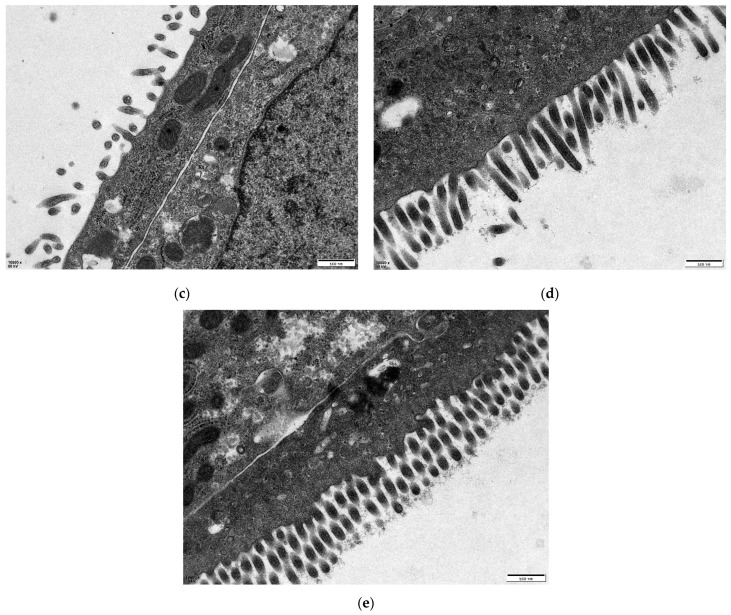
Morphological observation of Caco-2 cell monolayer model. (**a**) The planar microvilli structure of caco-2 cells under an electron microscope on the 7th day of modeling; (**b**) the planar microvilli structure of caco-2 cells under an electron microscope on the 15th day of modeling; (**c**) the vertical microvilli structure to the cell surface under transmission electron microscopy on the 7th day of modeling; (**d**) the vertical microvilli structure to the cell surface under transmission electron microscopy on the 15th day of modeling; (**e**) the vertical microvilli structure to the cell surface under transmission electron microscopy on the 20th day of modeling.

On the 7th, 15th, and 20th days of cell culture, the samples were examined using a scanning electron microscope and transmission electron microscope. The results are presented in Figure 5. It can be observed that on the 7th day, a considerable number of microvilli with a relatively loose structure began to differentiate on the cell surface, accompanied by noticeable intercellular. On the 15th day, the microvilli increased in number and arranged more closely. By the 20th day, no intercellular gaps were observed. The apical side was vertically covered with neat microvillus structures, forming a dense brush border. This level of differentiation reached its peak state resembling villus structures found in the small intestine, indicating that the cells were suitable for subsequent transport tests.

### 3.4. Bioavailability of Total I and As

#### 3.4.1. The Relationship between Bioavailability of Total I and As and Transport Time

**Figure 6:**
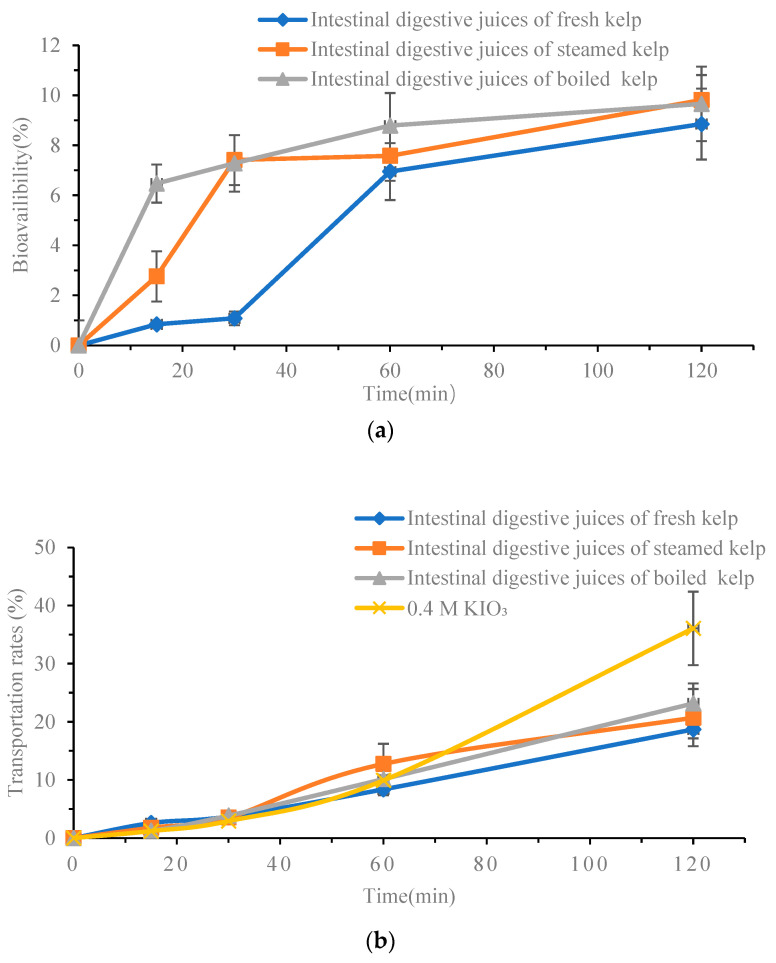
Bioavailability of I and As with time. Values are mean ± standard error (*n* = 3). (**a**) Bioavailability of I with time; (**b**) bioavailability of As with time.

In this study, the bioavailability of iodine and arsenic in the intestinal digestive juices of cooking/uncooked kelp was investigated, along with their transport properties over time in the Caco-2 monolayer. Inorganic iodine (KIO_3_) at the same concentration was used as a control for inorganic iodine. Figure 6 shows that the permeability of iodine and arsenic in the Caco-2 monolayer of samples increased with the extension of time. Initially, the transport rate was low for the first 30 min, but it increased gradually after that. After 2 h, the bioavailability of As in intestinal digestive juice of raw kelp, steamed kelp samples, and boiled kelp samples was 8.85%, 9.81%, and 9.66%, respectively. The bioavailability of I in the three groups was 18.71%, 20.72% and 23.18%, respectively. The bioavailability of I in 0.4 M KIO_3_ solution was 31.84%. These results indicated that cooking treatment could enhance the iodine absorption in the intestine. Furthermore, it suggested that the transport efficiency of inorganic iodine was higher than that of kelp iodine in the intestine.

While the bioavailability calculated by transportation rates provides valuable information, it does not fully reflect the absolute content of element intake, which is a crucial parameter for comparative analysis. Therefore, the bioavailable contents of different groups were computed to facilitate comparative analysis. The results are presented in Table 4. It can be observed that cooking treatment increased the bioavailability of iodine and arsenic. However, the bioavailable contents of the two elements were significantly reduced. After 2 h transportation, the bioavailable content of iodine in the raw materials intestinal digestive part was 55.56 ± 4.64 μmol/L, while that in the boiling kelp intestinal digestive part was 6.88 ± 1.01 μmol/L. Compared with the raw material group, the KIO_3_ control group exhibited twice as much bioavailable concentration of iodine. The results of the bioavailable concentration of arsenic were also consistent with iodine, and the lowest bioavailable content of arsenic was 210.43 ± 48.55 nmol/L in the intestinal digestive part of boiled kelp.

#### 3.4.2. Bioavailability of Total I with Concentration

**Figure 7:**
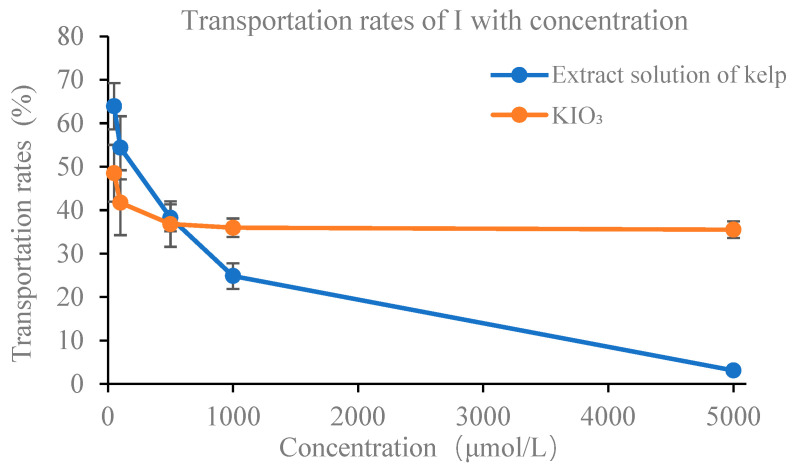
Transport of iodine with concentration. Values are mean ± standard error (*n* = 3).

The above results indicated that there were obvious disparities in the absorption and transport of kelp iodine and inorganic iodine in the Caco-2 monolayer. To further explore the bioavailability of kelp iodine and inorganic iodine at different concentrations, experimental groups with varying concentrations were set up. Figure 7 shows a gradual decrease in the transportation rates of iodine from kelp as the concentration increases, ranging from 63.93% to 3.14%. At a concentration of 500 μmol/L, the transport rates of both iodine in kelp and inorganic iodine exhibited similar transport rates, measuring at approximately 38.25% and 36.79%, respectively. At a concentration of 5000 μmol/L, the transport rate of iodine in kelp was only 3.14%. With the increase in concentration, the transport rate of KIO_3_ also decreased to a certain extent, but basically stabilized at around 35% with the concentration higher than 5000 μmol/L.

The transport efficiency of kelp iodine and KIO_3_ in monolayer cells is shown in Figure 7. The effective concentrations of different groups were analyzed and the results are presented in Table 5. At a concentration of 50 μmol/L, the biologically effective concentrations of iodine from kelp and KIO_3_ were as low as 31.96 ± 5.29 μmol/L and 24.26 ± 2.76 μmol/L. With increasing concentration, the biologically effective concentration of iodine showed a moderate increase. When the iodine concentration was below 500 μmol/L, the biologically effective concentration of iodine in kelp exceeded that of the KIO_3_ group. Specifically, at the concentration of 500 μmol/L, the biologically effective concentration of iodine extracted from kelp was 191.23 ± 30.54 μmol/L, while that for KIO_3_ was 183.95 ± 28.32 μmol/L. The biologically effective concentration of inorganic iodine was found to be higher than that of iodine extracted from kelp. When the concentration of iodine exceeded 500 μmol/L, the biologically effective concentration of inorganic iodine maintained a high growth trend, whereas for iodine in kelp, it reached its maximum (248.56 ± 58.61 μmol/L) at an iodine concentration of 1000 μmol/L and then gradually declined. At a concentration of 5000 μmol/L, the biologically effective concentration of iodine extracted from kelp was 156.96 ± 34.22 μmol/L, significantly lower than that of the inorganic iodine group. These findings indicated notable differences in the absorption and transportation mechanisms between kelp-derived and inorganic forms of iodine as concentrations vary, which necessitated further exploration.

## 4. Discussion

Bioaccessibility refers to the proportion of the contents that can be dissolved in the gastrointestinal environment, representing the maximum amount of a test substance that could be absorbed by the body and serving as an indication of its maximum oral bioavailability [39]. The concept of bioavailability has been expressed in various ways in the literature. From a pharmacological perspective, bioavailability is defined as the proportion of the total amount of contents ingested via oral pathways that can pass through the gastrointestinal tract and eventually enter the systemic circulation [40]. While closely related, it is important to note that bioaccessibility and bioavailability are not equivalent. The absorption of efficiency and utilization of elements from different sources within the gastrointestinal system vary. The effective utilization of various elements in the edible state after food processing is essential for evaluating the safety of food consumption. This study investigates the absorption and utilization of iodine and arsenic in kelp by combining bioaccessibility and bioavailability measurements in both raw and cooked kelp, using a simulated gastrointestinal digestion system with an intestinal epithelial cell model.

Insufficient or excessive iodine intake could cause certain harm to the human body. in response to the World Health Organization’s recommendation for global iodine supplements, numerous countries and organizations have conducted research on appropriate iodine intake. These studies have employed various methods including cell experiments, animal experiments and population surveys of consumers to investigate the bioavailability of iodine in seaweed. While kelp is known for its high iodine content, it contains diverse forms of iodine, including both inorganic (I^−^ and IO_3_^−^) and organic forms (such as 3-iodo-L-tyrosine, MIT and 3,5-diiodo-L-tyrosine, DIT). Inorganic forms account for approximately 90% of total iodine content, while organic forms constitute around 10% [41,42]. Norwegian scholars studied the bioavailability of iodine in *Saccharina latissima* using Wistar rats and found that female Wistar IGS rats exhibited lower bioavailability of kelp-derived iodine compared to potassium iodide (KI), with values ranging from 5% to 6% and 19% to 27%, respectively. Additionally, the research revealed that high doses of kelp-derived iodine had no significant impact on circulating thyroid stimulating hormone (TSH) and free triiodothyronine (FT3) levels [43]. Spanish scholars utilized simulated gastrointestinal digestion combined with a Caco-2 monolayer cell transport model to explore the bioaccessibility and bioavailability of iodine in brown algae. The results showed that the bioaccessibility was 49–82% and the bioavailability was 2–7%. The data also showed that compared with other brown algae, the iodine concentration was higher in kombu, but the cell transport rates were lower [44]. A case–control study conducted by Korean scientists investigated 117 thyroid cancer patients, and 173 control patients underwent cancer screening. The study revealed a negative association between dietary iodine intake and thyroid cancer [45]. A scholar in Japan conducted a double-blind study on the consumption of kelp powder in 50 subjects and found no increase in serum thyroid hormone concentration after iodine supplementation from kelp [46]. While there are reports on the bioavailability of As in aquatic products, limited information is available regarding algal products. Previous research on the bioavailability of As in seaweed mainly focused on the estimation through in vitro digestion methods. Laparra discovered that the bioaccessibility of total arsenic increased significantly after cooking from 67.2% to 79.9% in *Porphyra* sp. and from 62.3% to 65.7% in *H. fusiforme*. Meanwhile, the bioaccessible content was significantly decreased after cooking, which aligns with our findings [23]. A research in Spain used Kombu as a model to perform a mass balance study for evaluating the accuracy of the bioavailability study of cooked seaweed. The results indicated that the concentration of arsenic decreased to one-third after cooking [47]. These findings suggest that while kelp contains high levels of I and As, their respective bioavailabilities differ significantly. Therefore, it is unscientific to judge food safety solely based on element content alone. Instead, considering bioavailability could serve as a reference for allowing high consumption of iodine from seaweeds [48].

The common cooking methods of kelp, including blanching, boiling, steaming, and stir-frying simmering, could reduce the content of elements of materials, especially soluble elements [40]. The loss rate depends on the ingredient type an cooking method. Previous studies have demonstrated that different processing techniques significantly decrease iodine levels in seaweed products: 10% through washing, 34–44% through soaking, and 58% through steaming and boiling [49]. This study investigates the impact of various cooking methods on the iodine and arsenic content in kelp. The findings reveal that boiling for 10 min leads to a significant reduction in both iodine and arsenic in kelp, with a loss of over 94% and 74%, respectively. The dissolution rates of iodine (80.21%) and arsenic (61.37%) within the gastrointestinal system align with previous research [43]. It has also been reported that the effects of blanching and salting treatment reduce different forms of iodine content in kelp, sorting by loss: I^−^ >IO_3_^−^ > MIT > DIT. Notably, the inorganic iodine ion (I^−^) loss rate was the highest at about 58.6% with blanching treatment [50]. While cooking enhances the bioaccessibility of iodine and arsenic in fresh kelp, it ultimately leads to a substantial decrease in their absolute contents. The bioavailability of iodine and arsenic in boiled kelp is notably lower than in fresh kelp, indicating that cooking significantly improves the edible safety of kelp. Furthermore, the study highlights differences in transport characteristics between iodine in kelp and inorganic iodine, suggesting that even when consumed in large quantities, kelp iodine is absorbed less efficiently. Overall, knowledge of the content of total iodine in kelp is insufficient. It is necessary to determine the fraction that is bioavailable for humans as well as consider variations caused by different cooking treatments and chemical forms of iodine (organic or inorganic). The vitro studies have demonstrated that the bioavailability of iodine from kelp is significantly lower than that from inorganic iodine (IO_3_^−^), which has implications for determining the maximal amount of kelp consumption. The biologically effective concentration of digested and absorbed iodine by the body from fresh kelp amounts to only 0.18~1.83% of its total content, indicating a significant improvement regarding its safety under edible conditions. Our findings support the view that the bioavailability of iodine from seaweeds can be considered safe for allowing high consumption due to their higher edible safety as an alternative source of supplemental dietary iodine compared to KIO_3_.

In summary, for a scientific evaluation of the “risk–benefit” issue concerning iodine and arsenic in seaweed, it is necessary to fully consider the biologically effective concentration, chemical forms, bioavailability, and dietary exposure of potentially toxic elements in seaweed under edible conditions. Merely assessing the total amount of elements is insufficient. The significance of this study is not only to explore the bioavailability of iodine and arsenic in kelp but also to reveal the transport characteristics of iodine in kelp with transit time and concentrations in the intestine, as well as the transportation differences between iodine from kelp and KIO_3_. This research provides scientific support for evaluating the edible safety of kelp products while also establishing a theoretical foundation for further revealing the absorption and transport mechanism of iodine in kelp.

## Figures and Tables

**Table 1:** Treatment of digestion methods of PBET and UBM.

Digestion Methods	Treatments	
PBET [26,27]	Gastric	Each group of samples was supplemented with 60 mL of standard pepsin solution, and the sample–enzyme mixture was incubated in a shaker at 37 °C and 150 rpm for digestion of 1 h. NaHCO_3_ was used to finish digestion by adjusting the sample to pH = 7.0. Subsequently, 10 mL of the digested samples were subjected to centrifugation at a speed of 4000 rpm for 10 min followed by filtration. The filtrate was collected as gastric digests.
	Intestinal	The remaining samples were supplemented with 0.03 g of trypsin and 0.10 g of bile salt, dissolved and incubated on a shaker for 4 h. Subsequently, the samples were subjected to centrifugation at a speed of 4000 rpm for 10 min followed by filtration. The filtrate was collected as intestinal digests.
UBM [28]	Oral	The samples of each group were added to 20 mL of standard saliva and incubated at 37 °C for 5 min.
	Gastric	The mixture above was supplemented with 30 mL of standard gastric simulator and placed in a shaker at 37 °C, with an agitation speed of 150 rpm for a duration of 1 h. NaHCO_3_ was used to finish digestion by adjusting the samples to pH = 7.0. Subsequently, a volume of 10 mL from each sample was subjected to centrifugation at a speed of 4000 rpm for 10 min followed by filtration. The filtrate was collected as gastric digests while the remaining residue proceeded to the intestinal digestion stage.
	Intestinal	The residue was mixed with 80 mL of a standard small intestine simulation solution and the pH was adjusted to 7.0 using NaHCO_3_. Subsequently, the mixture was placed in a shaker for 4 h. The samples were subjected to centrifugation at a speed of 4000 rpm for 10 min followed by filtration. The filtrate was collected as intestinal digests.

**Table 2:** Bioaccessibility of total I and As in raw/cooked kelp (%).

Bioaccessibility	Samples	I	As
PBET	Raw materials	59.86 ± 5.66 ^B^	48.16 ± 3.38 ^B^
	Steamed processing	65.49 ± 4.80 ^AB^	53.03 ± 4.68 ^B^
	Boiled processing	68.03 ± 5.28 ^A^	59.91 ± 5.79 ^A^
UBM	Raw materials	64.63 ± 4.60 ^B^	50.73 ± 4.11 ^B^
	Steamed processing	69.53 ± 6.34 ^B^	57.74 ± 4.74 ^A^
	Boiled processing	80.21 ± 6.97 ^A^	61.37 ± 5.01 ^A^

Values expressed as mean ± standard deviation (*n* = 3). Different capital letters in the same column indicated significant differences (*p* < 0.05).

**Table 3:** Content of total I and As in bioaccessible fractions.

Digestion Model	Samples	I (μg/L)		As (μg/L)	
		Gastric Phase	Intestinal Phase	Gastric Phase	Intestinal Phase
PBET	Raw materials	3188.20 ± 312.03 ^A^	580.29 ± 79.19 ^A^	32.49 ± 2.93 ^A^	5.53 ± 0.85 ^A^
	Steamed processing	1641.78 ± 90.55 ^B^	294.81 ± 51.40 ^B^	28.71 ± 2.54 ^B^	4.41 ± 0.53 ^B^
	Boiled processing	317.00 ± 23.34 ^C^	59.51 ± 7.10 ^C^	18.08 ± 1.73 ^C^	2.62 ± 0.46 ^C^
UBM	Raw materials	3505.51 ± 258.06 ^A^	563.31 ± 57.54 ^A^	34.38 ± 3.33 ^A^	5.66 ± 1.10 ^A^
	Steamed processing	1859.32 ± 167.60 ^B^	196.71 ± 20.40 ^B^	31.54 ± 2.76 ^B^	4.52 ± 0.63 ^B^
	Boiled processing	372.52 ± 33.17 ^C^	71.42 ± 6.34 ^C^	18.47 ± 1.51 ^C^	2.74 ± 0.29 ^C^

Values expressed as mean ± SD (*n* = 3). Different capital letters in the same column indicated significant differences in bioaccessible fractions between different treatment groups (*p* < 0.05).

**Table 4:** Bioavailable content of I and As in gastrointestinal digestive juices of kelp.

Elements	Time/Min	15	30	60	120
I (μmol/L)	Raw materials	7.65 ± 1.39 ^Ad^	11.01 ± 1.93 ^Ac^	24.90 ± 3.07 ^Bb^	55.56 ± 4.64 ^Ba^
	Steamed processing	2.72 ± 0.76 ^Bc^	5.42 ± 1.49 ^Cc^	19.47 ± 5.27 ^Cb^	31.62 ± 7.52 ^Ca^
	Boiled processing	0.38 ± 0.17 ^Cd^	1.14 ± 0.14 ^Dc^	3.01 ± 0.43 ^Db^	6.88 ± 1.01 ^Da^
	0.4 M KIO_3_	3.66 ± 0.64 ^Bc^	8.79 ± 2.18 ^Bc^	29.77 ± 3.92 ^Ab^	108.25 ± 19.00 ^Aa^
As (nmol/L)	Raw materials	42.47 ± 7.78 ^Bc^	54.81 ± 13.67 ^Cc^	353.24 ± 58.14 ^Ab^	449.72 ± 71.99 ^Aa^
	Steamed processing	121.91 ± 7.78 ^Ac^	327.46 ± 51.40 ^Ab^	334.93 ± 36.03 ^Ab^	433.50 ± 61.59 ^Aa^
	Boiled processing	122.58 ± 18.04 ^Ac^	129.02 ± 14.59 ^Bc^	167.03 ± 24.72 ^Bb^	210.43 ± 48.55 ^Ba^

Note: Different capital letters in the same line indicated significant differences in bioavailability between different treatment groups; different lowercase letters in the same column indicated significant differences in biological availability at different times in the group (*p* < 0.05).

**Table 5:** Bioavailable content of extracted iodine from kelp and KIO_3_ (μmol/L).

Concentration (μmol/L)	Extracted Iodine from Kelp	KIO_3_
50	31.96 ± 5.29	24.26 ± 2.76
100 *	54.37 ± 3.94	41.73 ± 7.01
500	191.25 ± 30.54	183.95 ± 28.32
1000 *	248.56 ± 58.61	359.49 ± 60.37
5000 *	156.96 ± 34.22	1776.26 ± 240.20

Note: * indicates that there is a significant difference between the biologically effective concentration of iodine extracted from kelp and inorganic iodine at the same concentration, *p* < 0.05.

## Data Availability

The original contributions presented in the study are included in the article, further inquiries can be directed to the corresponding author.
